# Atomic-scale thermal manipulation with adsorbed atoms on a solid surface at a liquid-solid interface

**DOI:** 10.1038/s41598-019-49677-x

**Published:** 2019-09-13

**Authors:** Kunio Fujiwara, Masahiko Shibahara

**Affiliations:** 1 Center for Atomic and Molecular Technologies, Osaka University, 2-1 Yamadaoka, Suita, Osaka 565-0871 Japan; 2 Department of Mechanical Engineering, Osaka University, 2-1 Yamadaoka, Suita, Osaka 565-0871 Japan

**Keywords:** Nanoscience and technology, Applied physics

## Abstract

Modulating thermal transport through interfaces is one of the central issues in nanoscience and nanotechnology. This study examined thermal transport between atoms adsorbed on a solid surface and a liquid phase based on non-equilibrium molecular dynamics. The heat flux was detected at sub-atomic spatial resolution, yielding a two-dimensional map of local heat flux in the vicinity of the adsorbed atoms on the surface. Based on the detected heat flux, the possibility of atomic-scale thermal manipulation with the adsorbed atoms was examined by varying the interaction strengths between the liquid molecules and atoms adsorbed on the surface. The results of the local heat flux at the single-atom scale clearly showed effects of the adsorbed atoms on the thermal transport through the liquid-solid interface; they can significantly enhance the heat flux at the single-atom scale using degrees of freedom normal to the macroscopic temperature gradient. The effect was especially evident for a low wettability surface, which provides key information on local enhancement at the single-atom scale of the thermal transport through a liquid-solid interface.

## Introduction

Modulating thermal transport through interfaces is one of the central issues in nanoscience and nanotechnology^1–3^. The thermal transport through interfaces has been intensively studied at the nanoscale by experiments^4–8^ and simulations^9–30^. In particular, practical control of the thermal transport through liquid-solid interfaces has been investigated, for instance, by modifying the wettability of the solid surface^31,32^, and by changing roughness or geometries of the solid surface^14,16,33^. Elucidating the mechanism and characteristics of thermal transport through the liquid-solid interface is important not only for applications of such functional surfaces but also for nanofluidic devices.

Recent advances in scanning probe microscopy (SPM) enables us to manipulate an atom on a solid surface using SPM^34–39^. For example in reference^36^, the authors demonstrated the selective removal of an adatom on a Si(111)-(7 × 7) surface using atomic force microscopy (AFM). An atom adsorbed on a surface is the fundamental structure of a solid surface, and it may be possible to control the thermal transport through interfaces at the single-atom scale. In general, the thermal transport through a liquid-solid interface is enhanced for high wettability surfaces, and low wettability surfaces show higher thermal resistance^40^. Changing the properties of an atom adsorbed at a liquid-solid interface may allow thermal manipulation at the smallest length scales. Especially for a low wettability surface with high thermal resistance, this manipulation is expected to enhance the local thermal transport through the liquid-solid interface at the single-atom scale. Such thermal manipulation is a key method for commutation and switching of heat flow through the interface at the atomic scale, and a fundamental understanding of this thermal transport is crucially important to interfacial transport phenomena in physics, chemistry, and biology. However, a clear picture of the role of an atom adsorbed on a solid surface to the thermal transport has not yet been elucidated, due to lack of techniques to experimentally detect or model heat flux at the single-atom sale.

Molecular dynamics simulation has helped elucidate the mechanisms behind interfacial thermal transport, and is becoming a standard tool for predicting thermal transport at interfaces based on microscopic information^9–30^. However, previous studies have not been able to provide a picture of heat flow at the single-atom scale in an interfacial region; the heat flux detected in space was limited to a single-dimensional representation along a temperature gradient^11,16^, which was a bottleneck to understanding thermal transport mechanisms more precisely based on molecular physics. Our recent study^41^ detected heat flux at the atomic scale as a two-dimensional map in a liquid-solid interfacial region, and directional heat flux was observed between contacting solid and liquid layers in the interfacial region. Although controlling the directional quality of the heat flux is the ultimate control of interfacial thermal transport, such thermal manipulation based on the directional heat flux has not been attempted. In the present study, we conducted non-equilibrium molecular dynamics simulations to reveal the roles of atoms adsorbed on a solid surface in the thermal transport through a liquid-solid interface. The heat flux was detected at sub-atomic spatial resolution, allowing the construction of a two dimensional map of the local heat flux in the vicinity of the adsorbed atoms on the solid surface. Based on the detected heat flux, we explored the possibility of atomic-scale thermal manipulation by varying the interaction strengths between liquid molecules and atoms of the solid. We focused on the local enhancement of the thermal transport through a low wettability surface at the single-atom scale, and evaluated the thermal transport through the adsorbed atoms at a liquid-solid interface.

## Methods

A microscopic expression of the energy transport equation for monatomic particles is given by^41–44^,

1 $$\begin{array}{rcl}{\bf{j}}({\bf{r}}) & = & \langle {\bf{j}}({\bf{r}},t)\rangle ,\\  & = & \langle \frac{1}{{\Omega }_{{\bf{r}}}}[\sum _{i\in {\Omega }_{{\bf{r}}}}{e}_{i}{{\bf{v}}}_{i}+\frac{1}{2}\sum _{i}\sum _{j\ne i}{{\bf{r}}}_{ij}^{\ast }({{\bf{v}}}_{i}\cdot {{\bf{F}}}_{ij})]\rangle .\end{array}$$

Here, the local and instantaneous energy flux **j**(**r**, *t*) is defined in the local volume Ω_**r**_, and the net energy flux can be obtained as the time-averaged expression: ${\bf{j}}({\bf{r}})=\langle {\bf{j}}({\bf{r}},t)\rangle$. Equation (1) is valid for the systems in which particles interact based on two body potentials. In Eq. (1), the force and velocity of the particles are denoted as **F** and **v**, respectively. The subscripts *i* and *j* denote the *i*th and *j*th particles in the system, respectively, and the energy of the *i*th particle with the mass *m*_*i*_ and potential energy *ϕ*_*ij*_ is defined as ${e}_{i}=(1/2){m}_{i}{{\bf{v}}}_{i}^{2}+(1/2){\Sigma }_{j}{\varphi }_{ij}$. It should be noted that r^*^ is a line segment included in the local volume Ω_**r**_. Although the methodology to obtain the local quantities was limited to a single-dimension, in this study, we calculated heat flux in three-dimensional space at sub-atomic resolution in a liquid-solid interfacial region, and obtained a two-dimensional map of the local heat flux based on our previous study^41,44^. The energy transport from a solid atom to liquid molecules at the liquid-solid interface can be obtained based on the second term on the right hand side in Eq. (1), the inner product **v** **·** **F**, which can be expressed by the component representation:

2 $$\begin{array}{rcl}{j}_{i}^{atm} & = & \langle \,{j}_{i}^{atm}(t)\rangle =\sum _{j(\ne i)\in l}\langle {{\bf{v}}}_{i}\cdot {{\bf{F}}}_{ij}\rangle ,\,\,i\in s,\\ {j}_{i,xy}^{atm} & = & \sum _{j(\ne i)\in l}\langle {v}_{i,x}{F}_{ij,x}+{v}_{i,y}{F}_{ij,y}\rangle ,\\ {j}_{i,z}^{atm} & = & \sum _{j(\ne i)\in l}\langle {v}_{i,z}{F}_{ij,z}\rangle ,\end{array}$$

in which ${j}_{i}^{atm}$ represents the energy transfer from the *i*th solid atom to the liquid phase, defined as the component along the temperature gradient in this study. In Eq. (2), **F** represents the force between the liquid molecules and solid atoms, and *l* and *s* denote the liquid and solid respectively. The terms ${j}_{i,xy}^{atm}$ and ${j}_{i,z}^{atm}$ are the contributions to ${j}_{i}^{atm}$ by the *xy* and *z* components of the velocity and force vector, respectively, which were used to evaluate the heat flux through the interface in detail. As described below, the *z* component corresponds to the temperature gradient direction, which is normal to the interface through which heat flows, and the *xy* component represents the tangential components in our calculation system.

**Figure 1:**
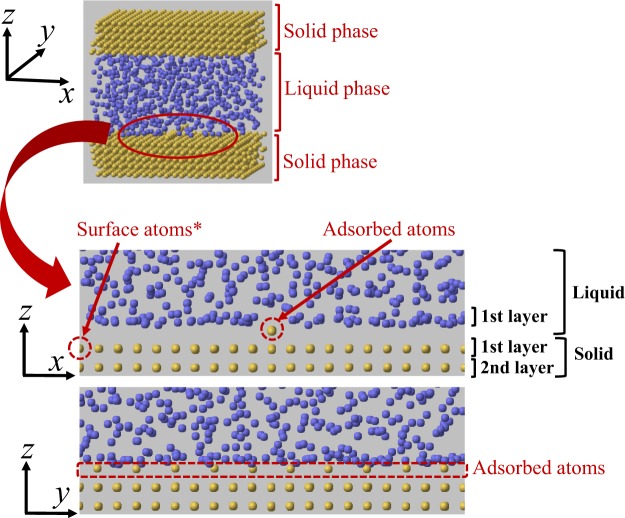
Calculation model with adsorbed atoms. The adsorbed atoms exist at the center of the system on the lower solid surface in the *x-z* plane. It can be seen that the adsorbed atoms consist of 10 atoms and are arranged in the *y* direction. In this study, the atoms of the solid, except for the adsorbed atoms, are the surface atoms; however we focus on the atoms indicated as Surface atoms* in Fig. 1 at the boundary of the system (x^*^ = 0.0) when we calculate physical quantities from the surface atoms.

To represent the liquid-solid interfacial system, we adopted a modelled system in which liquid molecules exist between two solid walls, and heat flows from the lower to the upper solid wall through the liquid-solid interfaces in the *z* direction. Liquid molecules were represented by argon, while platinum atoms were used as the atoms making up the solid. A periodic boundary condition was applied in the *x* and *y* directions, and the system size *L*_*x*_ × *L*_*y*_ × *L*_*z*_ was set as 40 × 40 × 39Å^3^. A subset of platinum atoms was adsorbed on the lower solid wall as shown in Fig. 1, where 10 adsorbed atoms were arranged in the *y* direction. The adsorbed atoms exist at the center of the system on the lower solid surface in the *x-z* plane. After the relaxation calculation, the energy in the calculation system is stable, and the adsorbed atoms shown in Fig. 1 don’t move around on the solid surface during the simulations. The surface atoms indicate the solid atoms except for the adsorbed atoms; however we focused on the atoms at the boundary in the modelled system (see Fig. 1) to calculate the thermal transport from the surface atoms, which also consisted of 10 atoms in the *y* direction. The interaction between the liquid molecules and that between the atoms of the solid (including the adsorbed and surface atoms) were both assumed to be described by the 12-6 Lennard-Jones (LJ) potential:

3 $$\varphi ({r}_{ij})=4\varepsilon [{(\sigma /{r}_{ij})}^{12}-{(\sigma /{r}_{ij})}^{6}],$$

where *r*_*ij*_ is the distance between the *i*th and *j*th particles. In this study, reduced units were used based on the standard parameters of Ar molecules (*σ* = 3.405 Å, *ε* = 1.67 × 10^−21^ J, and mass), and the reduced parameters used for the solid-solid interactions were *σ*_*ss*_ = 0.746 and *ε*_*ss*_ = 65.39^45^. The interactions between the liquid molecules and surface solid atoms (the atoms of the solid except for the adsorbed atoms) were also described using a potential of the same form with a constant parameter *ε*_*s*_:

4 $${\varphi }_{ls}({r}_{ij})=4{\varepsilon }_{s}[{({\sigma }_{ls}/{r}_{ij})}^{12}-{({\sigma }_{ls}/{r}_{ij})}^{6}],$$

where *σ*_*ls*_ was calculated using the Lorentz-Berthelot rule. For the interactions between the liquid molecules and atoms adsorbed on the solid, we used the function:

5 $${\varphi }_{ls}({r}_{ij})=4{\varepsilon }_{a}[{({\sigma }_{ls}/{r}_{ij})}^{12}-{({\sigma }_{ls}/{r}_{ij})}^{6}],$$

with the parameter *ε*_*a*_. The values of the interaction strengths *ε*_*a*_ and *ε*_*s*_ were varied from 0.5 to 2.0, which represents varying the interface wettability from low to high^46^. The upper and lower solid walls consisted of five atomic layers with the fcc(100) surfaces facing the liquid, and the gap between the solid walls was set to be 7.0. The temperature of the solid walls was controlled using the Langevin method^47^ at the fourth layers from the liquid region, fixing the temperature of the fifth atomic layers of the solid at the lower and upper walls. The temperatures of the upper and lower solid surfaces were set to be T^*^ = *T*/(*ε*/*k*_*B*_) = 0.41 and 1.2, respectively, where *k*_*B*_ is the Boltzmann constant.

## Results

**Figure 2:**
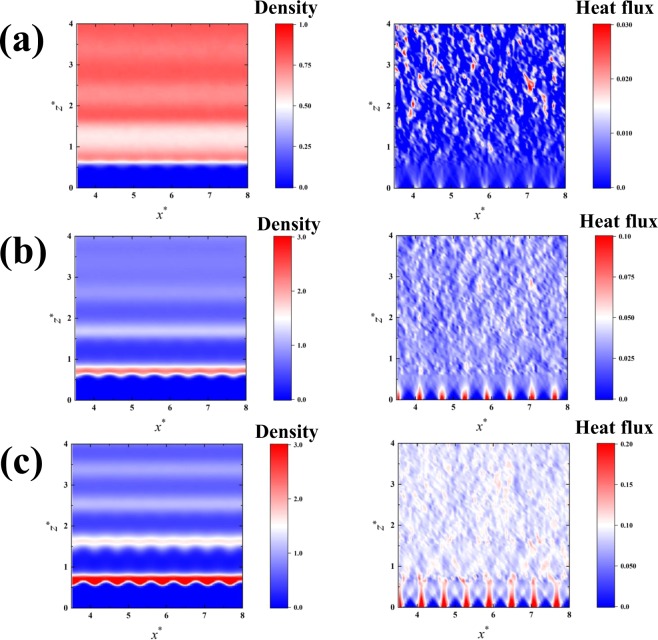
Two-dimensional density distribution of liquid molecules, and local heat flux from the solid atoms to the liquid phase in the cases of the systems without adsorbed atoms: (**a**) *ε*_*s*_ = 0.1, (**b**) *ε*_*s*_ = 0.5, and (**c**) *ε*_*s*_ = 1.0. The results are the averaged values for 100000000 time steps, and obtained at a resolution of dx^*^ × dz^*^ = 0.059 × 0.059. The first layer of the surface solid atoms is located at z^*^ = 0.0, and the first layer of the liquid phase exists approximately at z^*^ = 0.7.

First, we focus on the systems without adsorbed atoms, and show density distributions of liquid molecules and two-dimensional maps of the heat flux (*z* component) detected at sub-atomic resolutions based on Eq. (1) in Fig. 2. The results are good references to understand the local heat flux from the adsorbed atoms later. Here, the results were normalized using the LJ parameters of argon. The local quantities were calculated at a spatial resolution dx^*^ × dz^*^ = 0.059 × 0.059 in the *x*-*z* plane, and obtained as the values averaged over 1.0 × 10^8^ time steps. The first layer of the solid atoms facing the liquid phase is located at z^*^ = 0.0, and the first liquid layer exists approximately at 0.7. Note that the heat flux is detected even in the space where there are no atoms between the first solid and liquid layers by calculating the local quantities based on Eq. (1). The results clearly show the directional heat fluxes at the atomic scale, especially in the immediate vicinity of the solid surface, and which is remarkable in the cases of the surface with high wettability as confirmed in Fig. 2(b,c). The results are consistent with our previous study^41^ (low pressure condition). The heat fluxes detected correspond to the density results of the liquid molecules in the vicinity of the solid surface, which suggests that the liquid structure in the vicinity of the surface is important to understand the local heat flux at atomic scale.

**Figure 3:**
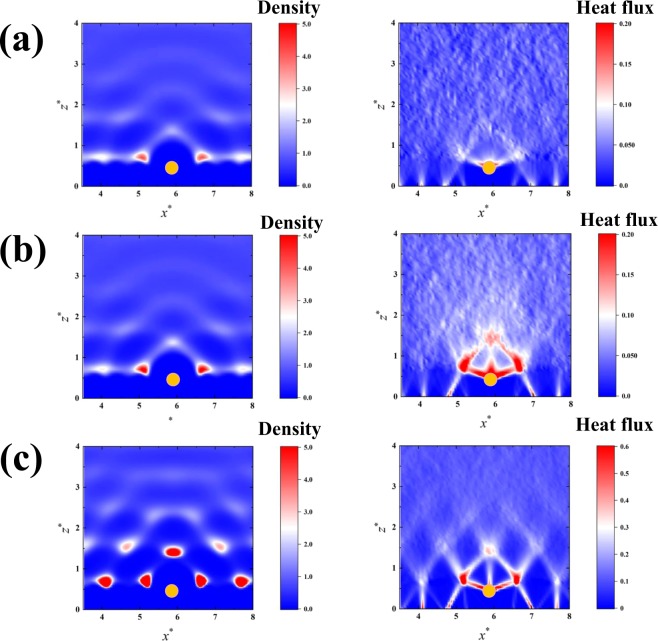
Two-dimensional density distribution of liquid molecules, and local heat flux from the solid atoms to the liquid phase in the cases of the systems with adsorbed atoms: (**a**) (*ε*_*a*_, *ε*_*s*_) = (0.5, 0.5), (**b**) (*ε*_*a*_, *ε*_*s*_) = (2.0, 0.5) and (**c**) (*ε*_*a*_, *ε*_*s*_) = (2.0, 1.0). The yellow circles indicate the adsorbed atoms. The results are the averaged values for 100000000 time steps, and obtained at a resolution of dx^*^ × dz^*^ = 0.059 × 0.059. The first layer of the surface solid atoms is located at z^*^ = 0.0.

Next, we show the results obtained in the systems with adsorbed atoms. Figure 3 shows density distributions of liquid molecules and two-dimensional maps of the heat flux (*z* component) detected at sub-atomic resolutions based on Eq. (1), in the vicinity of the atoms adsorbed on the solid surface. The adsorbed atoms are located at (x^*^, z^*^) = (5.9, 0.49), and indicated as the yellow circles in Fig. 3. The first layer of the solid atoms facing the liquid phase is located at z^*^ = 0.0. The typical cases in which the wettability of the adsorbed atoms is higher than or equal to that of the surface atoms (*ε*_*a*_ ≥ *ε*_*s*_) are shown in Fig. 3. The results of the liquid density show characteristic density distributions in the vicinity of the adsorbed atoms depending on the parameters *ε*_*a*_ and *ε*_*s*_. Furthermore, the results clearly show the directional heat fluxes from the adsorbed atoms to the liquid phase at single-atom scale, corresponding to the density distributions of liquid molecules, especially for relatively high values of *ε*_*a*_ and *ε*_*s*_ (Fig. 3(b,c)). This indicates that the enhancement of the heat flux from the adsorbed atoms to the liquid phase is due to the liquid density around the adsorbed atoms. The two-dimensional heat fluxes with directionalities detected from the adsorbed atoms in Fig. 3, suggest that the adsorbed atoms transfer the heat from the surface solid atoms to the liquid phase using a degree of freedom normal to the macroscopic temperature gradient.

**Figure 4:**
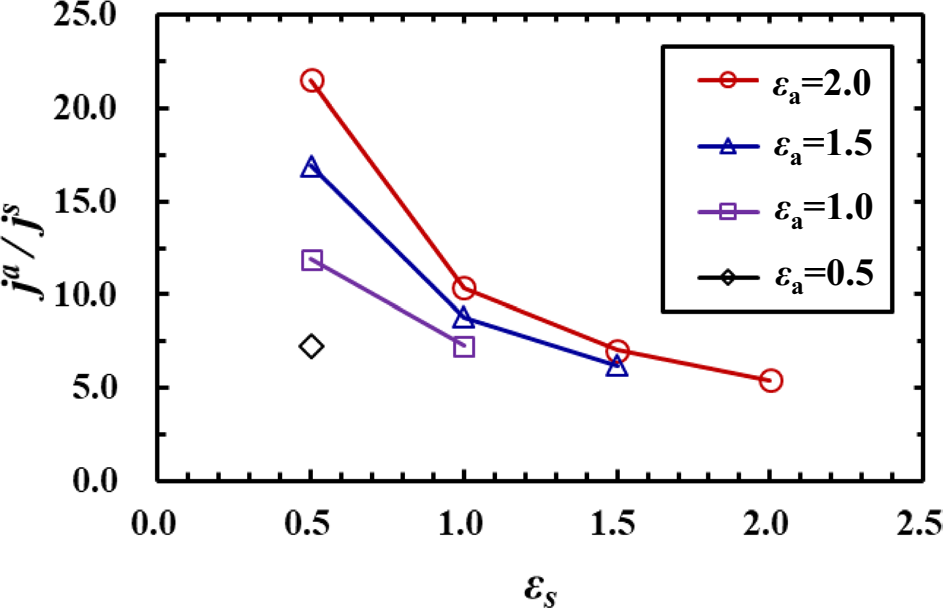
Ratio of the thermal transport from the adsorbed atoms *j*^*a*^ to that from the surface solid atoms *j*^*s*^ for various values of *ε*_*a*_ and *ε*_*s*_. The values of *j*^*a*^ and *j*^*s*^ were calculated based on Eq. (2), and *j*^*s*^ was obtained from the atoms indicated as “Surface atoms*” in Fig. 1.

**Figure 5:**
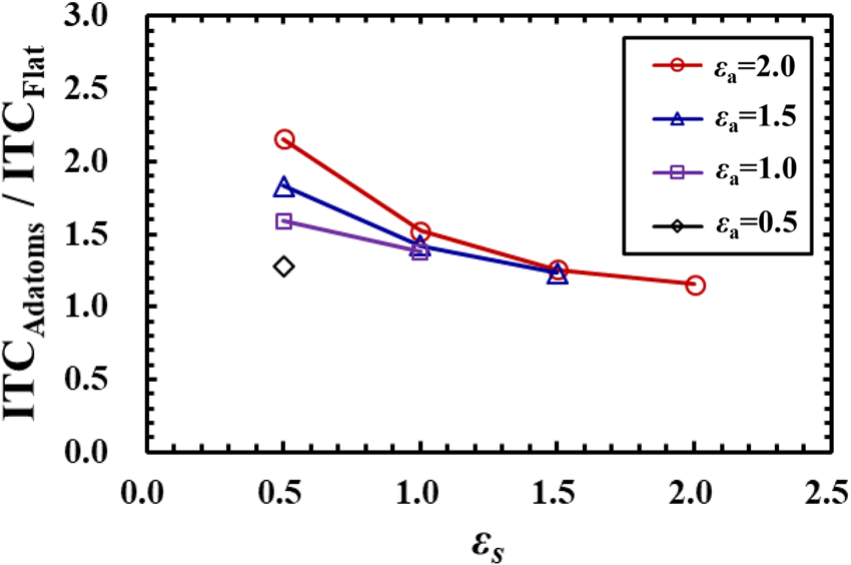
Ratio of the interfacial thermal conductance of the surface with adsorbed atoms (ITC_Adatoms_) to that without adsorbed atoms (ITC_Flat_). The parameters of *ε*_*a*_ and *ε*_*s*_ represent the wettability conditions for the adsorbed and surface atoms, respectively. The ITC is calculated using the overall heat flux across the interface, and the temperature jump is evaluated at the plane where the 1st layer of the solid atoms facing the liquid phase exist.

Figure 4 shows the ratio of the thermal transport from an adsorbed atom to that from a surface solid atom for various values of *ε*_*a*_ and *ε*_*s*_. In Fig. 4, *j*^*a*^ and *j*^*s*^ denote the thermal transport in the *z* direction from the adsorbed and surface atoms, respectively, which were each calculated as an average over 10 atoms. The values of *j*^*a*^ and *j*^*s*^ were calculated based on Eq. (2), and *j*^*s*^ was obtained from the atoms indicated as “Surface atoms*” in Fig. 1. The cases in which the wettability of the adsorbed atoms is higher than or equal to that of the surface atoms (*ε*_*a*_ ≥ *ε*_*s*_) are shown in Fig. 4, and the cases of *ε*_*a*_ = *ε*_*s*_ mean the same wettability condition for the adsorbed and surface atoms. The results indicate that thermal transport from the adsorbed atoms is higher than that from the surface atoms, and is significantly enhanced especially when the wettability of the surface solid atoms is low (*ε*_*s*_ = 0.5). Over 5 times higher values were observed even in the cases of *ε*_*a*_ = *ε*_*s*_ in this study, which shows that the adsorbed atoms are a crucial element for the thermal transport through the liquid-solid interface. As a reference data, we present results of the interfacial thermal conductance (ITC) in Fig. 5. The ITC of the surface with adsorbed atoms (ITC_Adatoms_) and that without adsorbed atoms (ITC_Flat_) were calculated using the temperature jump at the interface and the overall heat flux across the liquid-solid interface. The temperature jump at the interface was evaluated at the plane where the 1st solid atoms facing the liquid phase exist. Figure 5 shows the ratio of the ITC_Adatoms_ to the ITC_Flat_ in the cases of *ε*_*a*_ ≥ *ε*_*s*_. The results clearly reveal that the ITC_Adatoms_ shows higher values compared with the ITC_Flat_, and the effect is remarkable for the low wettability surfaces, especially by using the adsorbed atoms with high wettability. It should be noted that the ITC calculated by using the overall heat flux across the interface depends on the density and configurations of the adsorbed atoms on the solid surface, which means we can enhance the overall ITC putting more adsorbed atoms on the solid surface. In the present study, we put individual atoms separately on a flat solid surface as a line in the *y* direction, to investigate the local heat flux at the single-atom scale enhanced by the adsorbed atoms, to show possibilities of manipulation of the heat flux at the single-atom scale.

**Figure 6:**
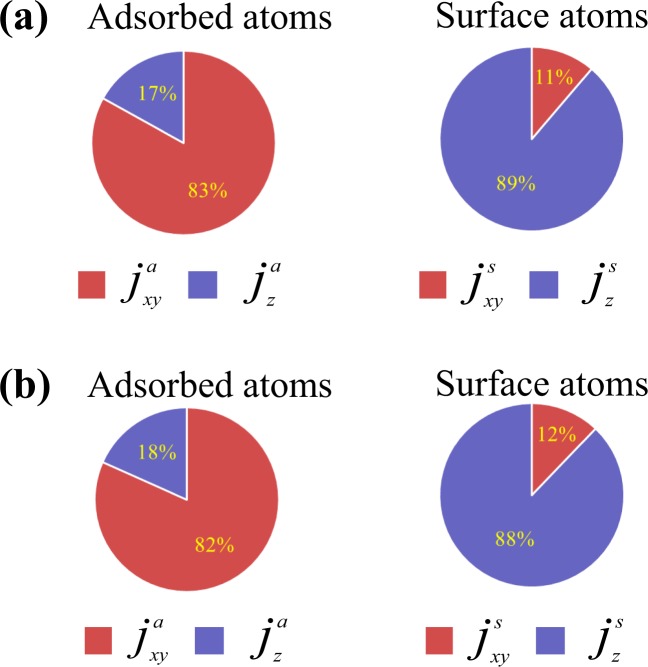
Contributions of *xy* and *z* components to the thermal transport from the adsorbed atoms and from the surface atoms to the liquid phase in the cases of (*ε*_*a*_, *ε*_*s*_) = (0.5, 0.5) and (*ε*_*a*_, *ε*_*s*_) = (2.0, 0.5). Here, *j*^*a*^ and *j*^*s*^ denote the thermal transport from the adsorbed and surface atoms, respectively. The values were obtained based on Eq. (2), and the *xy* and *z* components correspond to the directions normal and parallel to the temperature gradient, respectively.

Finally, we examine the contributions of the velocity and force components of the heat flows in the *z* direction through the adsorbed atoms in the cases of (*ε*_*a*_, *ε*_*s*_) = (0.5, 0.5) and (*ε*_*a*_, *ε*_*s*_) = (2.0, 0.5) in Fig. 6. Here, *j*^*a*^ and *j*^*s*^ denote the thermal transport from the adsorbed and surface atoms, respectively. The values of *j*_*xy*_ and *j*_*z*_ were obtained based on Eq. (2), and the *xy* and *z* components correspond to the directions normal and parallel to the macroscopic temperature gradient, respectively. The results clearly reveal that the contribution of the *xy* component is dominant for the adsorbed atoms, although the effect is weak for the surface solid atoms, which means that the adsorbed atoms enhance the thermal transport through themselves using the components normal to the macroscopic temperature gradient.

## Conclusions

We reported on the roles played by atoms adsorbed on a solid surface in thermal transport through a liquid-solid interface based on molecular dynamics, and explored atomic-scale thermal manipulation with the adsorbed atoms. Calculating the local heat flux in space with sub-atomic resolution at the liquid-solid interface, we clearly detected heat fluxes from the adsorbed atoms to the liquid phase, not only along the macroscopic temperature gradient direction, but also in the direction normal to the temperature gradient. The effect was significant, and distinct from that of the surface atoms. We conclude that this phenomenon enables the adsorbed atoms to enhance the thermal transport significantly at the single-atom scale, which suggests possibilities for atomically precise thermal manipulation at the liquid-solid interface especially by using the adsorbed atoms on a low wettability surface.

### Numerical details

The simulations were conducted using a time interval Δt^*^ =Δ *t* /( *σ*(*m*/*ε*)^1/2^ ) = 9.3 × 10^−4^, and the equation of motion was integrated using the velocity Verlet method. The dimensionless cut-off distance for the interactions was chosen to be 4.0. First, the temperature of the liquid was controlled at 0.83 using the velocity-scaling method for 1.0 × 10^5^ time steps, and the relaxation calculation was conducted for 1.0 × 10^7^ time steps without controlling the liquid temperature. Using the Langevin method during the simulations, the temperatures of the upper and lower solid surfaces were set to be T^*^ = *T*/( *ε*/*k*_*B*_ ) = 0.41 and 1.2, respectively, where *k*_*B*_ was the Boltzmann constant. A relatively large temperature gradient was imposed in the system, with the objective of detecting the local heat flux at the sub-atomic scale in the liquid-solid interfacial region. The averaged pressure (P^*^ = *P*/( *ε*/*σ*^3^)) was approximately 0.23 in each simulation. The local quantities were calculated at a spatial resolution dx^*^ × dz^*^ = 0.059 × 0.059 in the *x*-*z* plane, and obtained as the values averaged over 1.0 × 10^8^ time steps after the relaxation calculation.

## Data Availability

Output data is available from the corresponding author on reasonable request.
